# Circ_0020256 induces fibroblast activation to drive cholangiocarcinoma development via recruitment of EIF4A3 protein to stabilize KLF4 mRNA

**DOI:** 10.1038/s41420-023-01439-5

**Published:** 2023-05-13

**Authors:** Zongyan Li, Zuxiao Chen, Shiying Li, Xiangjun Qian, Lei Zhang, Guojie Long, Jiancong Xie, Xiaoming Huang, Zheyu Zheng, Weidong Pan, Haiyan Li, Dawei Zhang

**Affiliations:** 1 Department of Pancreatic Hepatobiliary Surgery, Department of General Surgery, The Sixth Affiliated Hospital, Sun Yat-sen University, Guangzhou, 510650 Guangdong Province P.R. China; 2 Department of Hepatobiliary Surgery, The Second Affiliated Hospital of Guangzhou Medical University, Guangzhou, 510260 Guangdong Province P.R. China; 3 Key Laboratory of Molecular Target & Clinical Pharmacology, School of Pharmaceutical Sciences, Guangzhou Medical University, Guangzhou, 511436 Guangdong Province P.R. China; 4 Department of Breast Surgery, Department of General Surgery, The Sixth Affiliated Hospital, Sun Yat-sen University, Guangzhou, 510650 Guangdong Province P.R. China

**Keywords:** Cancer, Diseases

## Abstract

Cancer-associated fibroblasts (CAFs) are a kind of stromal cells in the cholangiocarcinoma (CCA) microenvironment, playing crucial roles in cancer development. However, the potential mechanisms of the interaction between CCA cells and CAFs remain obscure. This work investigated the role of circ_0020256 in CAFs activation. We proved circ_0020256 was up-regulated in CCA. High circ_0020256 expression facilitated TGF-β1 secretion from CCA cells, which activated CAFs via the phosphorylation of Smad2/3. Mechanistically, circ_0020256 recruited EIF4A3 protein to stabilize KLF4 mRNA and upregulate its expression, then KLF4 bound to TGF-β1 promoter and induced its transcription in CCA cells. KLF4 overexpression abrogated the inhibition of circ_0020256 silencing in TGF-β1/Smad2/3-induced CAFs activation. Furthermore, CCA cell growth, migration, and epithelial-mesenchymal transition were favored by CAFs-secreted IL-6 via autophagy inhibition. We also found circ_0020256 accelerated CCA tumor growth in vivo. In conclusion, circ_0020256 promoted fibroblast activation to facilitate CCA progression via EIF4A3/KLF4 pathway, providing a potential intervention for CCA progression.

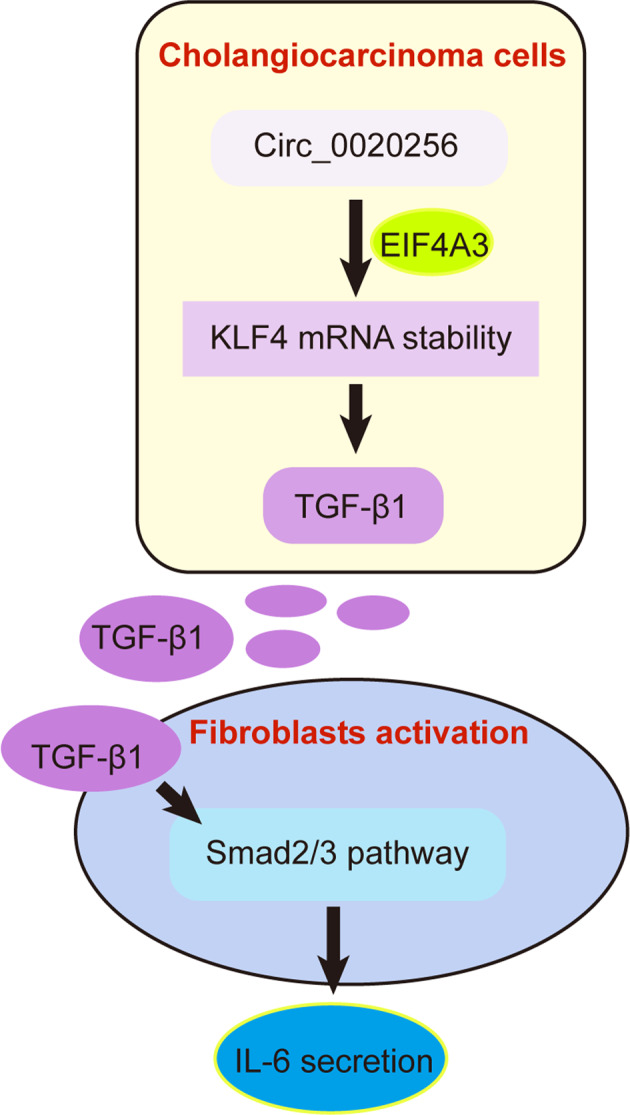

## Introduction

Cholangiocarcinoma (CCA), originated from biliary epithelial cells, is a fatal tumor with high metastatic capacity [1]. There is a rising trend in the incidence of CCA worldwide, especially in Asia [2]. Although improvement in early diagnosis and treatment has achieved, only ~10% of CCA cases can survive more than 5 years, and most patients will die within 1 year after diagnosis [3]. Thus, understanding the mechanisms of CCA tumorigenesis is conducive to identify new therapeutic methods. Recently, the activated fibroblasts, known as cancer-associated fibroblasts (CAFs), have been accepted to exert pivotal roles in CCA microenvironment, which facilitates the malignant capacities of CCA cells [4]. It has been reported a pro-invasive communication between CAFs and CCA cells [5]. Fibroblast activation can be triggered by mediators released from tumor cells and tumor microenvironment [6]. Additionally, mounting evidence has demonstrated that CAFs enhanced the aggressiveness of CCA cells via secreting multiple cytokines, including IL-6 [5]. Thongchot et al. reported that IL-6 secreted from CAFs conferred chemotherapy resistance of CCA patients via inhibiting autophagy [7]. Therefore, repressing fibroblast activation might be an efficient way to control CCA aggressiveness.

Circular RNAs (circRNAs) are non-coding RNAs composed of covalently closed continuous loops. Aberrant expression of circRNAs correlates with the progression of various malignancies, including CCA [8]. For example, circNFIB was found to delay CCA proliferation and metastasis through inactivating MEK1/ERK pathway [9]. Our study indicated that circ_0020256 released from tumor-associated macrophages facilitated malignant development of CCA [10]. So far, the involvement of circ_0020256 in fibroblast activation in CCA microenvironment has not been clarified.

Eukaryotic translation initiation factor 4A3 (EIF4A3), assigned to the DEAD box protein family, has been reported to function as an RNA-binding protein (RBP) to play key roles in cancer progression via affecting mRNA stabilization. A study revealed that EIF4A3 led to PVT1 stabilization to enhance circLMNB2 expression, which contributed to lung adenocarcinoma progression [11]. Other evidence demonstrated that EIF4A3 promoted glioblastoma tumorigenesis via the stabilization of LINC00680 and TTN-AS1 [12]. Bioinformatic analysis predicted that EIF4A3 was a potential RBP for circ_0020256. Furthermore, TCGA database indicated that EIF4A3 was up-regulated in CCA. However, the influence between circ_0020256 and EIF4A3 on CCA development remains underexplored. Krüppel-like factor 4 (KLF4) is a transcription factor implicated in different biological processes, including proliferation, cell cycle progression, apoptosis, and differentiation [13]. KLF4 exhibits tumor promotive roles in a series of tumors [14]. For CCA, up-regulation of KLF4 facilitated epithelial-mesenchymal transition (EMT) through activating AKT and ERK1/2 signaling [15]. Interestingly, RNA Interactome database predicated that EIF4A3 could bind to KLF4 mRNA, suggesting the potential regulation of EIF4A3 in KLF4 mRNA stability.

Transforming growth factor β1 (TGF-β1) pathway activation could trigger fibroblast activation and thereby contributing to CAFs production [16]. A previous study suggested that hyperactivation of TGF-β1/Smad signaling facilitated trans-differentiation of fibroblasts into CAFs in colorectal cancer microenvironment [17]. Notably, KLF4, as a transcription factor, was predicted to bind to the promotor of TGF-β1. Therefore, TGF-β1/Smad pathway might be involved in KLF4-mediated fibroblast activation in CCA aggressiveness.

In this research, we hypothesized that circ_0020256 recruited EIF4A3 protein to stabilize KLF4 mRNA, which promoted TGF-β1/Smad pathway-mediated fibroblast activation. On the other hand, the activated fibroblasts caused autophagy inhibition and malignant progression of CCA cells via secreting IL-6. Our study clarified the pivotal role of circ_0020256 in the interplay between CCA cells and CAFs, identifying circ_0020256 as a treatment target for CCA.

## Results

### CCA cells facilitated fibroblast activation via releasing TGF-β1

**Fig. 1:**
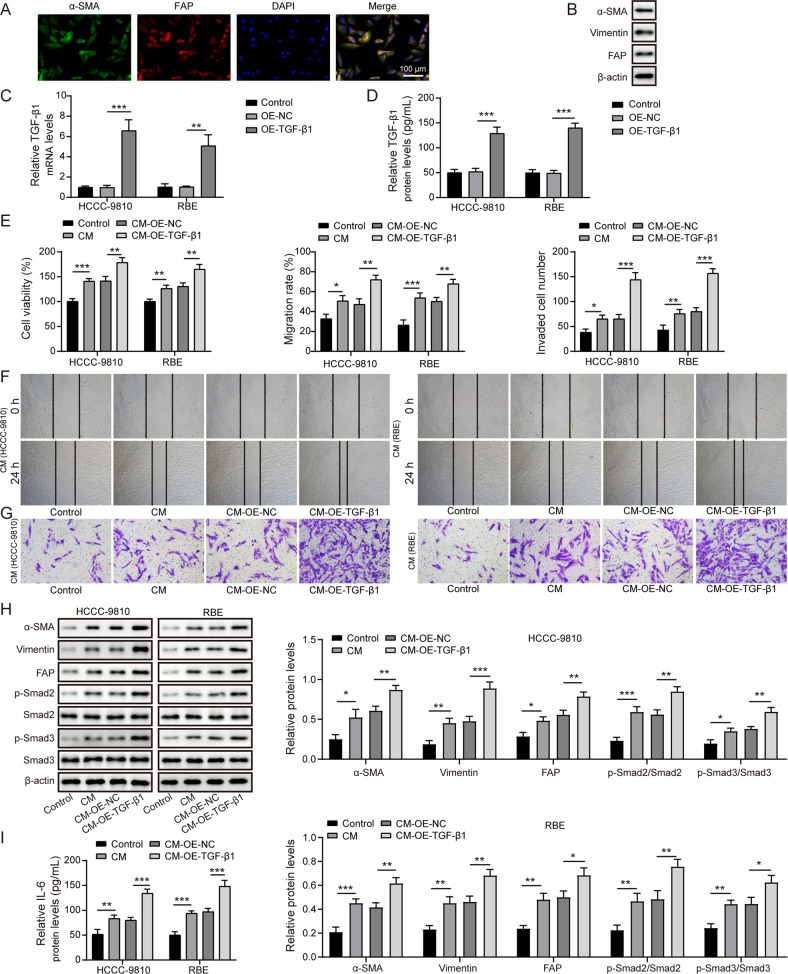
CCA cells-secreted TGF-β1 promoted fibroblast activation. **A** Immunofluorescence staining of α-SMA and FAP in CAFs isolated from CCA patients. **B** Western blotting analysis of α-SMA, vimentin, and FAP in primary CAFs. **C** CCA cells were transfected with OE-NC or OE-TGF-β1 and the mRNA expression of TGF-β1 was assessed by qRT-PCR. **D** Enzyme linked immunosorbent assay (ELISA) was adopted to determine TGF-β1 level in the supernatant of CCA cells. CAFs were treated with CM of CCA cells. **E** CAFs viability was detected by CCK-8. **F** Scratch assay for evaluation of migration ability of CAFs. **G** CAFs invasion was assessed by Transwell assay. **H** Western blotting analysis of α-SMA, vimentin, FAP, p-Smad2, Smad2, p-Smad3, and Smad3 levels in CAFs. **I** The protein level of IL-6 in the supernatant of CAFs was measured by ELISA. *P < 0.05, **P < 0.01, and ***P < 0.001.

First, CAFs were isolated from CCA tissues, α-SMA, vimentin and FAP expression in CAFs was validated by immunofluorescence staining (Fig. 1A) and western blotting (Fig. 1B). Furthermore, we overexpressed TGF-β1 in CCA cells by transfection OE-TGF-β1 plasmid (Fig. 1C). As expected, the release of TGF-β1 protein from TGF-β1-overexpressed CCA cells was enhanced (Fig. 1D). Next, CAFs were added with CM from CCA cells for 48 h. We found that the viability (Fig. 1E), migration (Fig. 1F), and invasion (Fig. 1G) of CAFs were promoted by CM treatment, which were further strengthened by CM collected from TGF-β1-overexpressed CCA cells. Moreover, CM remarkably up-regulated α-SMA, vimentin, FAP, p-Smad2, and p-Smad3 levels in CAFs (Fig. 1H). Similarly, CM from TGF-β1 overexpression group further enhanced the levels of these proteins (Fig. 1H). Notably, the release of IL-6 from CAFs was also increased after CM administration, which was more obvious in TGF-β1-overexpressed CM treatment group (Fig. 1I). These observations proved that CCA cells-secreted TGF-β1 promoted fibroblast activation and IL-6 production from CAFs.

### TGF-β1 stimulated fibroblast activation via activating Smad2/3 pathway

**Fig. 2:**
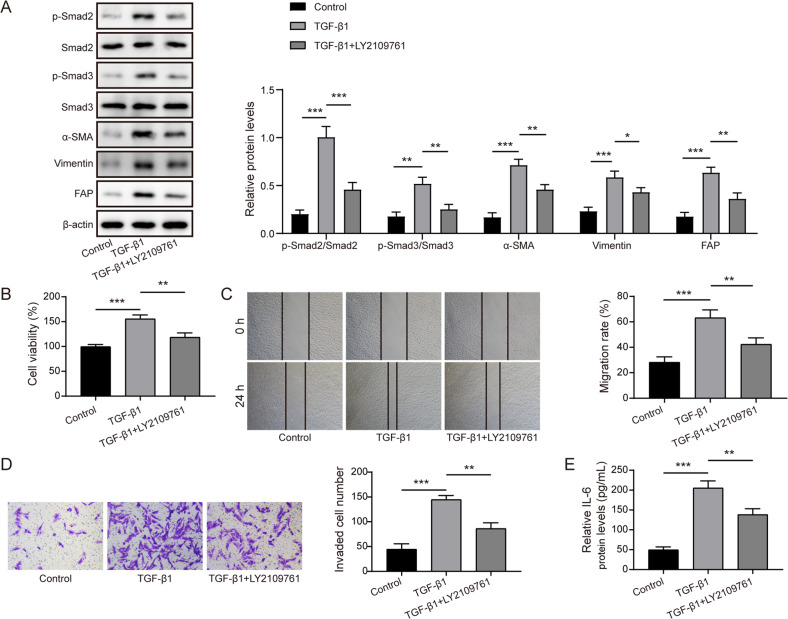
TGF-β1/Smad2/3 pathway activation was responsible for fibroblast activation. CAFs were treated with TGF-β1 (20 ng/mL) with or without LY2109761 (50 μM). **A** The protein abundance of α-SMA, vimentin, FAP, p-Smad2, Smad2, p-Smad3, and Smad3 in CAFs was determined by western blotting. **B** The viability of CAFs was detected by CCK-8. **C** The migration of CAFs was assessed by scratch assay. **D** The invasive ability of CAFs was evaluated by Transwell assay. **E** Enzyme linked immunosorbent assay (ELISA) detected the release of IL-6 protein from CAFs. *P < 0.05, **P < 0.01, and ***P < 0.001.

To verify the involvement of Smad2/3 pathway in TGF-β1-induced fibroblast activation, TGF-β1-stimulated CAFs were added with or without LY2109761. As illustrated in Fig. 2A, TGF-β1-induced up-regulation of p-Smad2, p-Smad3, α-SMA, vimentin, and FAP in CAFs was dramatically counteracted by LY2109761. In addition, the enhanced viability (Fig. 2B), migration (Fig. 2C), and invasion (Fig. 2D) in TGF-β1-treated CAFs were partly reversed by LY2109761. Besides, LY2109761 administration restrained the release of IL-6 from CAFs induced by TGF-β1 (Fig. 2E). Collectively, activation of Smad2/3 pathway was involved in TGF-β1-induced fibroblast activation.

### KLF4 transcriptionally activated TGF-β1 and enhanced its secretion from CCA cells

**Fig. 3:**
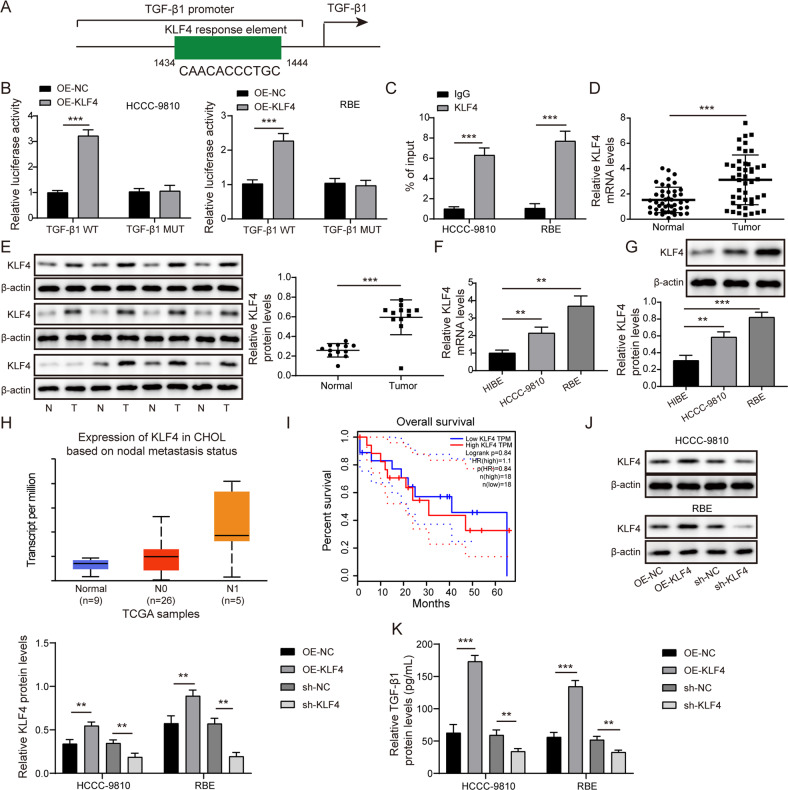
KLF4 transcriptionally activated TGF-β1 and enhanced its secretion from CCA cells. **A** The predicted binding sites of KLF4 to TGF-β1 promoter by JASPAR database. The direct binding between KLF4 and TGF-β1 promoter was validated by dual luciferase reporter assay (**B**) and ChIP (**C**) in CCA cells. qRT-PCR (**D**) and western blotting (**E**) analyses of KLF4 expression in paired CCA and non-tumor samples. KLF4 levels in CCA cells and normal HIBE cells were assessed by qRT-PCR (**F**) and western blotting (**G**). Bioinformatic analyses of the correlation between KLF4 expression and lymph node metastasis (**H**, UALCAN database) and survival of CCA patients (**I**, GEPIA database). **J** KLF4 protein level in OE-KLF4 or sh-KLF4-trasfected CCA cells was assessed by western blotting. **K** Enzyme linked immunosorbent assay (ELISA) for evaluation of TGF-β1 content in the supernatant of CCA cells. 45 paired patient samples were detected in **D** and 12 paired patient samples are randomly selected to test in **E**. **P < 0.01 and ***P < 0.001.

Next, the upstream mechanism of TGF-β1 in CCA cells was focused on. As predicted by JASPAR database, KLF4 possessed binding sites in TGF-β1 promoter (Fig. 3A). Moreover, overexpression of KLF4 raised the relative luciferase activity of TGF-β1-WT group, but not of TGF-β1-MUT group (Fig. 3B). Chromatin immunoprecipitation (ChIP) assay also confirmed the direct interaction between KLF4 protein and TGF-β1 promoter (Fig. 3C). Additionally, higher expression of KLF4 was found in CCA samples in comparison with controls (Fig. 3D, E). Consistently, KLF4 was also up-regulated in CCA cells in comparison with normal HIBE cells (Fig. 3F, G). Furthermore, high KLF4 expression strikingly correlated with high degree of lymph node metastasis and lower survival in CCA cases (Fig. 3H, I). To further explore the regulation of KLF4 in TGF-β1 level, CCA cells were transfected with OE-KLF4 or sh-KLF4 vector. The KLF4 overexpression and silence efficiencies were validated by western blotting (Fig. 3J). TGF-β1 secretion was evidently promoted in KLF4-overexpressed CCA cells, but restrained in KLF4-silenced cells (Fig. 3K). Taken together, KLF4 directly bound to TGF-β1 promoter to promote its expression and release from CCA cells.

### EIF4A3 protein interacted with KLF4 mRNA to enhance its expression in CCA cells

**Fig. 4:**
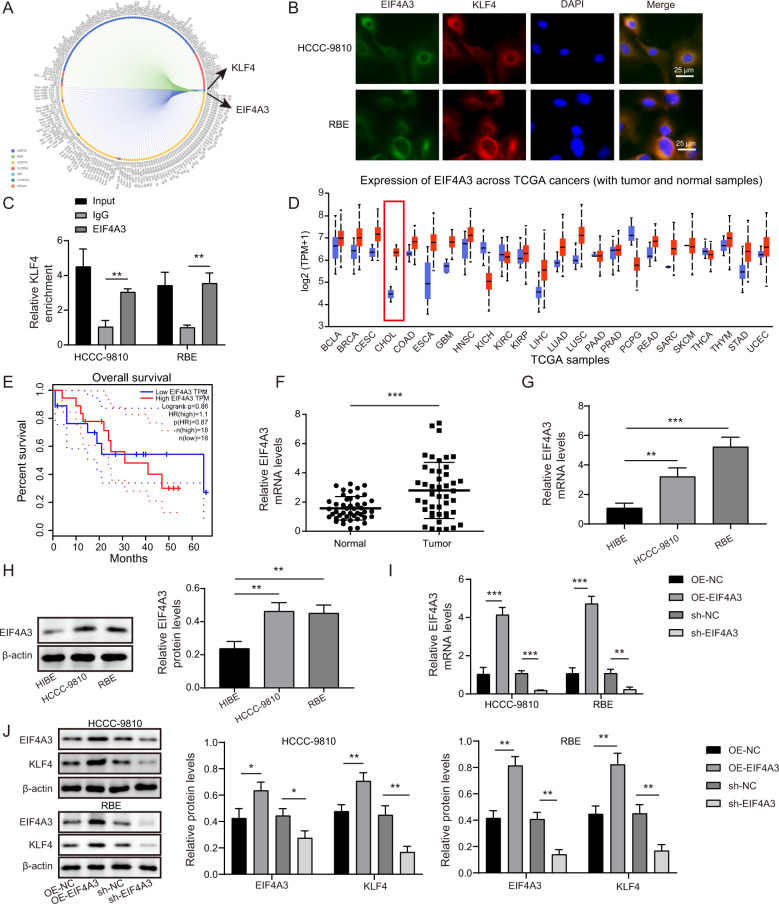
EIF4A3 protein interacted with KLF4 mRNA to enhance its expression in CCA cells. **A** The interaction between EIF4A3 protein and KLF4 mRNA was predicted by RNA Interactome database. **B** Co-localization of EIF4A3 protein and KLF4 mRNA in CCA cells was observed by immunofluorescence and FISH. **C** RIP assay confirmed the interplay between EIF4A3 protein and KLF4 mRNA. **D** UALCAN database showed up-regulation of EIF4A3 in CCA (also called CHOL) patients. **E** The correlation between EIF4A3 and survival of CCA patients was analyzed by GEPIA database. qRT-PCR analysis of EIF4A3 mRNA expression in CCA clinical samples (**F**) and cells (**G**). **H** Western blotting for EIF4A3 expression in CCA cells. The expression levels of EIF4A3 and KLF4 in CCA cells with various transfections were detected by qRT-PCR (**I**) and western blotting (**J**). 45 paired patient samples were detected in **F**. *P < 0.05, **P < 0.01, and ***P < 0.001.

To determine the potential modulatory mechanism of KLF4 expression, EIF4A3 was predicted as a potential RBP of KLF4 mRNA by RNA Interactome database (Fig. 4A). Fluorescence in situ hybridization (FISH) validated the co-localization of EIF4A3 protein and KLF4 mRNA in CCA cells (Fig. 4B). The interplay between EIF4A3 and KLF4 was further confirmed by RNA immunoprecipitation (RIP) assay (Fig. 4C). The UALCAN and GEPIA databases suggested that EIF4A3 was highly expressed in CCA (also called CHOL) samples (Fig. 4D), which suggested a lower survival of CCA cases (Fig. 4E). Moreover, the expression of EIF4A3 was obviously raised in CCA specimens (Fig. 4F) and cells (Fig. 4G, H) as compared with normal control groups. Furthermore, EIF4A3 overexpression distinctly enhanced KLF4 level in CCA cells, whereas EIF4A3 deficiency led to the opposite results (Fig. 4I, J). These data confirmed that EIF4A3 bound to KLF4 to increase its expression in CCA cells.

### Circ_0020256 recruited EIF4A3 protein to stabilize KLF4 mRNA

**Fig. 5:**
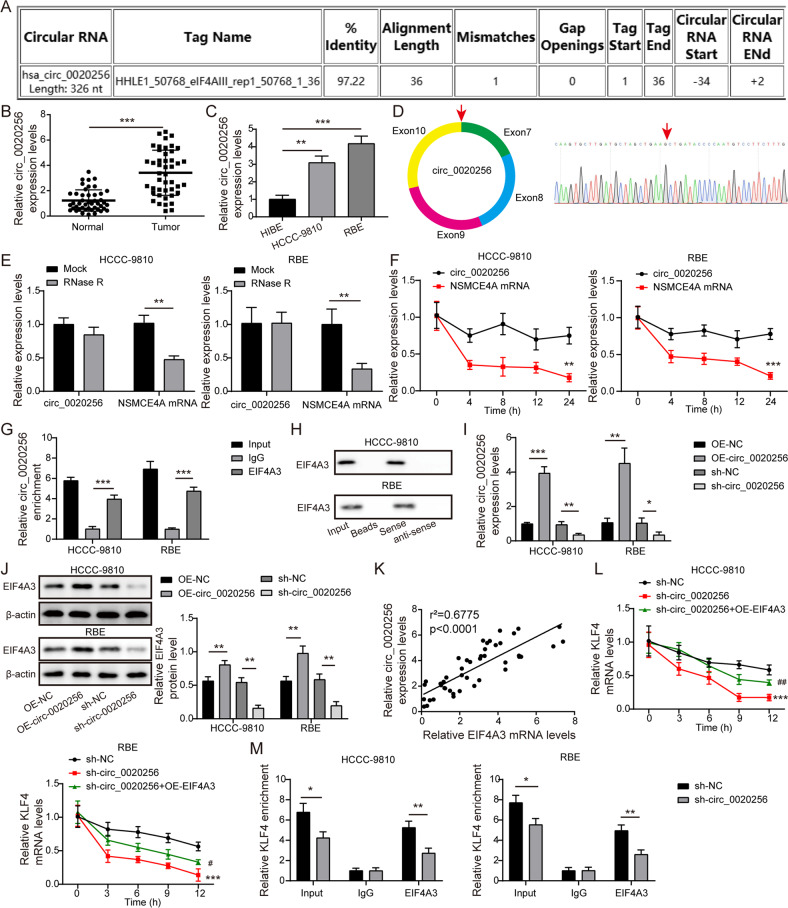
Circ_0020256 recruited EIF4A3 protein to stabilize KLF4 mRNA. **A** Circular RNA Interactome database analysis of the potential binding between circ_0020256 and EIF4A3 protein. Circ_0020256 expression in CCA specimens (**B**) and cell lines (**C**) was detected by qRT-PCR. **D** Schematic diagram of circ_0020256 formation and Sanger sequencing was used for identifying circ_0020256. The expression of circ_0020256 and linear NSMCE4A mRNA after exposure to RNase R (**E**) or actinomycin D (**F**) were assessed by qRT-PCR. The interaction between circ_0020256 and EIF4A3 protein was verified by RIP (**G**) and RNA pull-down assay (**H**). **I** qRT-PCR analysis of circ_0020256 level in CCA cells after transfection with OE-circ_0020256 or sh-circ_0020256. **J** EIF4A3 expression in CCA cells with different treatments was detected by western blotting. **K** The positive correlation between circ_0020256 and EIF4A3 expression in CCA samples. **L** The stability of KLF4 mRNA after exposure to actinomycin D was evaluated by qRT-PCR. **M** The interaction between KLF4 mRNA and EIF4A3 protein in circ_0020256-silenced CCA cells was determined by RIP assay. 45 paired patient samples were detected in **B** and **K**. *P < 0.05, **P < 0.01, and ***P < 0.001.

It has been documented that circ_0020256 exerted biological role in CCA [10]. Nonetheless, the effect of circ_0020256 on fibroblast activation in CCA microenvironment remains poorly understood. As analyzed by Circular RNA Interactome database, circ_0020256 possessed the potential to interact with EIF4A3 protein (Fig. 5A). In addition, high expression of circ_0020256 was verified in CCA (Fig. 5B, C). Subsequently, the “head-to-tail” back-splicing in circ_0020256 was validated by Sanger sequencing (Fig. 5D). Furthermore, circ_0020256 was resistant to RNase R or actinomycin D challenge, whereas its linear mRNA transcript NSMCE4A was unstable, indicating the closed-loop structure of circ_0020256 (Fig. 5E, F). Additionally, RIP detection verified a direct binding of EIF4A3 protein to circ_0020256 (Fig. 5G). RNA pull-down assay further demonstrated that EIF4A3 was remarkably aggregated in circ_0020256 pull-down complex (Fig. 5H). Besides, up-regulation of circ_0020256 raised EIF4A3 protein, whereas silence of circ_0020256 reduced EIF4A3 protein abundance (Fig. 5I, J). Circ_0020256 expression positively associated with EIF4A3 expression in CCA samples (Fig. 5K). The degradation of KLF4 in response to actinomycin D was reinforced by circ_0020256 depletion, and the promotive effect of circ_0020256 silencing on KLF4 degradation was reversed by EIF4A3 overexpression (Fig. 5L). RIP assay revealed that the interaction between EIF4A3 protein and KLF4 mRNA was weakened in circ_0020256-silenced CCA cells (Fig. 5M). To sum up, circ_0020256 contributed to stability of KLF4 mRNA via recruitment of EIF4A3 protein.

### Circ_0020256 stabilized KLF4 to induce fibroblast activation via activating TGF-β1/Smad2/3 pathway

**Fig. 6:**
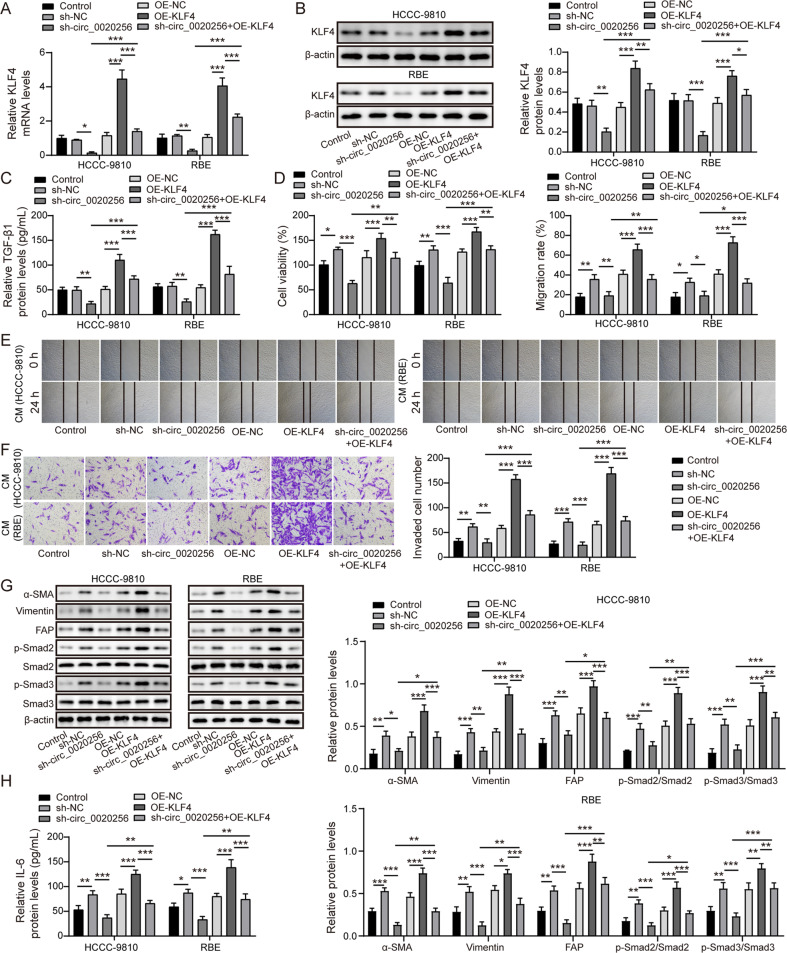
KLF4 was involved in circ_0020256-mediated fibroblast activation via activating TGF-β1/Smad2/3 pathway. KLF4 expression level in CCA cells from different groups was measured by qRT-PCR (**A**) and western blotting (**B**). **C** TGF-β1 level released from CCA cells was assessed by Enzyme linked immunosorbent assay (ELISA). **D** The results of CCK-8 assay for CAFs viability. **E** The migratory capacity of CAFs was determined by scratch assay. **F** The invasion of CAFs was assessed by Transwell assay. **G** The protein levels of α-SMA, vimentin, FAP, p-Smad2, Smad2, p-Smad3, and Smad3 in CAFs were evaluated by western blotting. **H** ELISA measured the release of IL-6 from CAFs. *P < 0.05, **P < 0.01, and ***P < 0.001.

To further determine whether circ_0020256 affected fibroblast activation in CCA microenvironment through modulating KLF4, CCA cells were transfected with sh-circ_0020256, OE-KLF4, or a combination of them, and their CM was collected. KLF4 level in CCA cells was declined after circ_0020256 knockdown, which was restored by KLF4 overexpression (Fig. 6A, B). In addition, circ_0020256 down-regulation repressed TGF-β1 secretion, whereas KLF4 overexpression facilitated TGF-β1 secretion and abolished sh-circ_0020256-mediated inhibitory effects (Fig. 6C). Moreover, the viability (Fig. 6D), migration (Fig. 6E), and invasion (Fig. 6F) of CAFs were restrained by CM derived from sh-circ_0020256 group, but promoted by CM derived from OE-KLF4 group. The inhibition in growth, migration and invasion of CAFs by circ_0020256-depleted CM was counteracted by KLF4 overexpression (Fig. 6D–F). Accordingly, the protein abundance of p-Smad2, p-Smad3, α-SMA, vimentin, FAP (Fig. 6G), and IL-6 protein release level (Fig. 6H) in CAFs were reduced by CM derived from circ_0020256-silenced CCA cells. As expected, treatment with CM from OE-KLF4 group obtained the contrary results and abrogated circ_0020256-depleted CM-induced above changes (Fig. 6G, H). These findings revealed that KLF4 was involved in circ_0020256-mediated fibroblast activation in CCA microenvironment.

### CAFs repressed autophagy to confer malignant capacities of CCA cells via IL-6 secretion

**Fig. 7:**
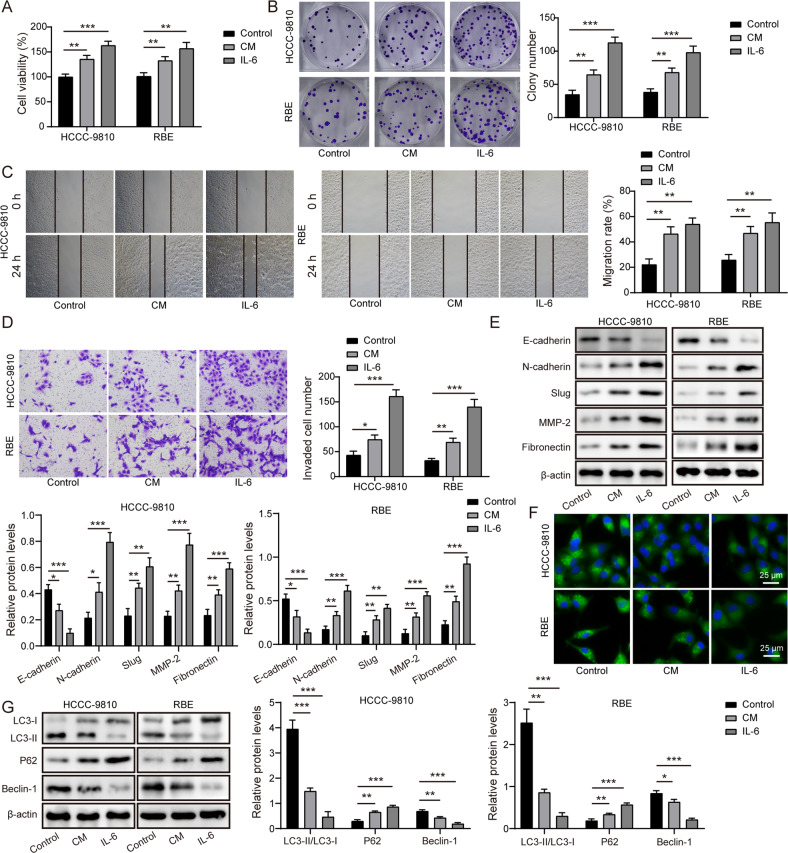
CAFs-derived IL-6 facilitated growth and migration of CCA cells via autophagy inhibition. CCA cells were treated with CAFs-derived CM or IL-6. CCA cell proliferation was evaluated by CCK-8 (**A**) and colony formation assay (**B**). **C** CCA cell migration was detected by scratch assay. **D** Transwell assay determined CCA cell invasion. **E** Western blotting analysis of E-cadherin, N-cadherin, slug, MMP-2 and fibronectin protein levels in CCA cells. **F** The images of immunofluorescence staining of LC3. **G** Western blotting analysis of LC3 II/I, p62, beclin-1 protein abundance. *P < 0.05, **P < 0.01, and ***P < 0.001.

To clarify the influence of CAFs-derived IL-6 on malignant phenotypes of CCA cells, we added CM collected from CAFs or IL-6 recombinant protein to CCA cells. The viability and colony formation of CCA cells were facilitated after administration with CAFs-derived CM or IL-6 (Fig. 7A, B). Furthermore, CAFs-derived CM or IL-6 treatment enhanced the migratory and invasive capacities of CCA cells (Fig. 7C, D). Moreover, EMT was promoted in CAFs-derived CM- or IL-6-treated CCA cells as confirmed by down-regulation of E-cadherin and up-regulation of N-cadherin, slug, MMP-2 and fibronectin (Fig. 7E). Then we found that treatment with CM of CAFs or IL-6 weakened immunofluorescence staining of LC3 in CCA cells (Fig. 7F). Consistently, LC3 II/I ratio and beclin-1 level were decreased, while p62 level was enhanced in CCA cells after CAFs-derived CM or IL-6 administration (Fig. 7G). The above results suggested that CAFs-derived IL-6 conferred malignant phenotypes of CCA cells via autophagy suppression.

### Circ_0020256 contributed to tumor growth and fibroblast activation in vivo

**Fig. 8:**
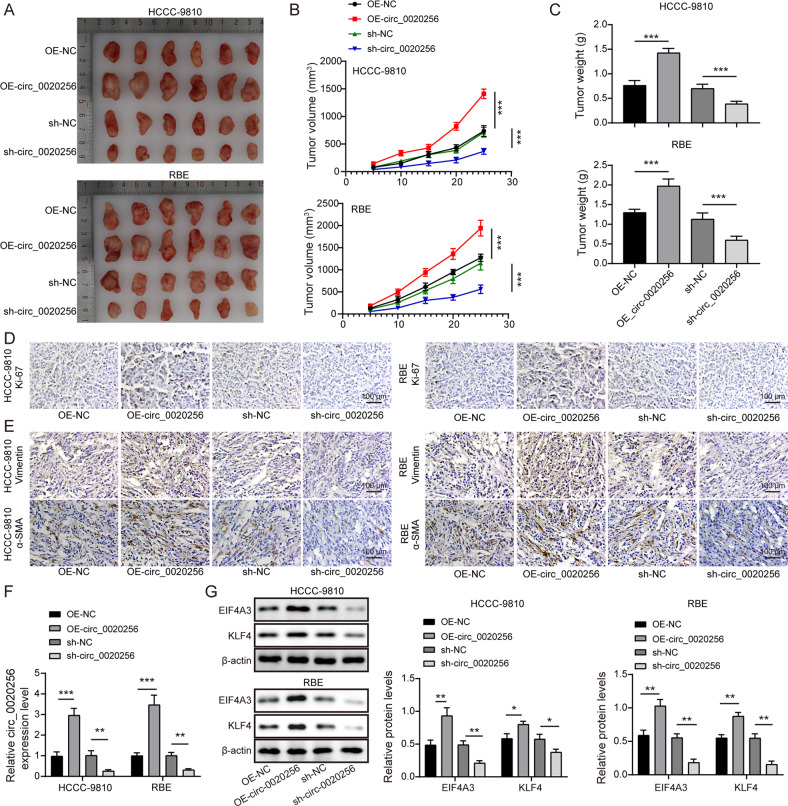
Circ_0020256 favored tumor growth in nude mice via the EIF4A3/KLF4 axis. **A** Images of the xenografts are shown. Tumor growth curve (**B**) and weight (**C**) are presented. **D**, **E** Immunohistochemical staining of Ki-67, vimentin, and α-SMA in tumors. **F** Circ_0020256 expression in xenograft tumor tissues was measured by qRT-PCR. **G** Western blotting analysis of EIF4A3 and KLF4 protein expression in tumors. *P < 0.05, **P < 0.01, and ***P < 0.001.

Finally, the role of circ_0020256 in tumor growth and fibroblast activation was explored in vivo. Remarkably, overexpression of circ_0020256 elevated the tumor weight and volume, however, opposite results were obtained when circ_0020256 was knocked down (Fig. 8A–C). Next, the expression levels of Ki-67 (Fig. 8D), and vimentin and α-SMA (makers of fibroblast activation, Fig. 8E) were raised in OE-circ_0020256 group, and these results were reversed in sh-circ_0020256 group. Moreover, EIF4A3 and KLF4 were highly expressed in circ_0020256-overexpressed tumors, but lowly expression in circ_0020256-silenced tumors (Fig. 8F, G). Hence, circ_0020256 drove transplanted tumor growth in the nude mice by modulating EIF4A3/KLF4 pathway.

## Discussion

CCA is a malignant tumor accompanied by the presence of high abundant activated CAFs [4]. Recently, the aggressive phenotypes of CAFs in CCA have been considered as important prognostic indicator [18], supporting that CAFs exert key roles for the aggressiveness of CCA. On the other hand, tumor cells facilitate fibroblast activation via secreting cellular factors [19]. Therefore, targeting the communication between CCA cells and CAFs may help aid for treating CCA patients. In this work, we demonstrated that circ_0020256 stimulated the activation of CAFs in CCA microenvironment to drive CCA progression. Mechanistically, circ_0020256 interacted with EIF4A3 protein to stabilize KLF4 mRNA, KLF4 transcription factor then directly bound to TGF-β1 promoter and promoted its transcription, which eventually activated Smad2/3 pathway in CAFs. In turn, the activated CAFs-secreted IL-6 to confer the malignant capacities of CCA cells via repressing autophagy. Our observations for the first time described the molecular mechanisms underlying the interplay between CCA cells and CAFs, providing potential therapeutic interventions for CCA.

TGF-β1 has been accepted as a pivotal mediator in the communication between cancer cells and stromal cells, which regulates growth, motility, and malignant transformation [20]. CAFs are suggested to be derived from TGF-β1-mediated myofibroblast transition of local fibroblasts [21]. The activated TGF-β1 pathway specifically triggers the phosphorylation of Smad2/3. Nan et al reported that pancreatic ductal adenocarcinoma cells-derived TGF-β1 resulted in CAFs activation through the Smad2/3 pathway [22]. In consistent with these studies, our data demonstrated that TGF-β1 released from CCA cells activated CAFs and enhanced their abilities of proliferation, migration, and invasion through Smad2/3 pathway activation. KLF4, a member of the Sp1 transcription factor family, is responsible for the regulation of gene expression [23]. Interestingly, mounting evidence has demonstrated that KLF4 functioned as a transcriptional regulator of TGF-β1, although the role of KLF4/TGF-β1 axis in CCA remains unclear. For instance, KLF4 exerted the oncogenic functions through transcriptional activation of TGF‐β1 to maintain stemness and mesenchymal capacities of colorectal cancer stem cells [24]. Li et al. [25] suggested that KLF4 delayed osteoarthritis progression via activation of TGF-β1 pathway. Here, KLF4 was found to be highly expressed in CCA. KLF4 overexpression transcriptionally activated TGF-β1 and enhanced its release from CCA cells. Thus, TGF-β1/Smad2/3 pathway-induced CAFs activation in CCA microenvironment was triggered by KLF4.

Recently, circRNAs have been demonstrated to interact with regulatory RBPs, and thereby affecting the mRNA stability of downstream target genes. For example, circRHOBTB3 was reported to inhibit colorectal cancer aggressiveness via regulating HuR-mediated PTBP1 mRNA stability [26]. Circ_0020256 derived from tumor-associated macrophages has been documented to contribute to CCA development [10]. However, the role of circ_0020256 in the communication between CCA cells and CAFs has not been illuminated. Our bioinformatic analysis found that circ_0020256 contained binding sites in EIF4A3 protein, which was confirmed by RIP and RNA pull-down assays. A previous study showed that EIF4A3 promoted the circular formation of circ-ZNF609 during CCA development [27]. EIF4A3, functioned as an RBP, is involved in the modulation of mRNA stability, and is suggested as a treatment target for a series of tumors. For example, circ_0068631 recruited EIF4A3 to enhance c-Myc mRNA stability, which accelerated growth and metastasis of breast cancer [28]. Of note, we validated that EIF4A3 could interact with KLF4 mRNA and promoted KLF4 expression. Furthermore, our data provided first evidence that circ_0020256 increased KLF4 mRNA stability by recruiting EIF4A3 protein, thus promoting CAFs activation to drive CCA development.

CAFs are stromal cells participated in the regulation of tumor progression [29]. Growing evidence has revealed that CAFs-derived cytokines function as “intermediates” for the interplay between CAFs and tumor cells [30]. IL-6 released by CAFs could drive the malignant development of CCA [31]. Autophagy is biological process that is responsible for the degradation of redundant cell components in lysosomes [32]. Defective autophagy has been considered to favor CCA development [33]. IL-6 could promote carcinogenesis via inhibition of autophagy [34]. Consistent with the previous observations, we found that treatment with CM from CAFs or IL-6 facilitated growth, metastasis, EMT, and autophagy inhibition of CCA cells. Therefore, our results indicated that the IL-6 release by activated CAFs in turn contributed to CCA progression via insufficient autophagy.

One limitation of our work lies in the limited sample size of CCA specimens, which might result in a statistical bias. Besides, whether the activated CAFs can favor CCA aggressiveness via up-regulating circ_0020256, and thereby forming a positive feed-back loop between CCA cells and CAFs, is another limitation. In addition, we used two commercially available CCA cell lines but not primary CCA cells isolated from CCA tissues to evaluate the effect on the activation of CAFs isolated from CCA tissues. The usage of primary CCA cells from CCA tissues may be more supportive of the conclusion than the commercially available CCA cell lines. These issues need to be further investigated. In conclusion, we first identified that circ_0020256 recruited EIF4A3 protein to stabilize KLF4 mRNA, which transcriptionally activated TGF-β1 and enhanced its secretion from CCA cells. In addition, CCA cells-derived TGF-β1 triggered CAFs activation and IL-6 release via Smad2/3 pathway, which in turn conferred the malignant capacities of CCA cells via autophagy inhibition. Our findings provide a theoretical basis for identifying novel interventions for CCA.

## Materials and methods

### Clinical samples

Forty-five pairs of fresh tumor tissues and tumor-adjacent normal tissues were obtained from CCA patients during surgery at the Sixth Affiliated Hospital, Sun Yat-sen University and were immediately put in the liquid nitrogen after acquisition. All enrolled patients did not receive any anti-tumor treatments and signed their informed consent. The clinicopathological characteristics of CCA cases were indicated in Supplementary Table 1. All experimental procedure was approved by the Ethics Committee of the Sixth Affiliated Hospital, Sun Yat-sen University.

### Cell culture

Human CCA cell lines HCCC-9810 and RBE were obtained from Procell Life Science & Technology Co.,Ltd. (Wuhan, China) and cultured in RPMI-1640 medium (Thermo Fisher Scientific, USA) containing 10% fetal bovine serum (FBS, Thermo Fisher Scientific). Human intrahepatic biliary epithelial cells (HIBE) were provided by ScienCell Research Laboratories (USA) and maintained in EpiCM (ScienCell) containing 10% FBS. All cell lines were authenticated using STR profiling and tested for no mycoplasma contamination.

### Primary culture of CAFs and treatment

Primary CAFs were separated from CCA samples as previously reported [35]. In brief, CCA tissue samples were cut into pieces and maintained in DMEM (Thermo Fisher Scientific) containing 20% FBS and basic fibroblast growth factor (1 ng/mL, MedChemExpress, USA) at 37 °C with 5% CO_2_. After removing the unattached cells, myofibroblast-like CAFs at passages 4–8 were adopted. The successful isolation of CAFs was identified by high expression of α-SMA, vimentin, and FAP. Primary CAFs were stimulated with TGF-β1 (20 ng/mL, MedChemExpress) for 48 h. For the inactivation of Smad2/3 pathway, CAFs were treated with 50 μM LY2109761 (MedChemExpress).

### Cell transfection

Overexpression plasmid (OE) for TGF-β1 (OE-TGF-β1), OE-KLF4, OE-EIF4A3, OE-NC, short hairpin RNA for KLF4 (sh-KLF4), sh-EIF4A3, and sh-negative control (sh-NC) were provided by GenePharma (Shanghai, China). CCA cells were transfected with the above segments using Lipofectamine 2000 (Thermo Fisher Scientific).

### Lentivirus infection

Lentiviral particles carrying full-length human circ_0020256 or sh-circ_0020256 were produced by GenePharma. To obtain stably circ_0020256-overexpressing or silencing CCA cells, HCCC-9810 and RBE cells were infected with lentiviruses and selected with puromycin (Sigma-Aldrich, USA).

### Immunofluorescence staining

After fixation with 4% paraformaldehyde, cells were treated with 0.2% Triton X-100 and blocked in 2% BSA for 1 h. Then, incubation with primary antibodies against α-SMA (1:500, ab124964, Abcam, UK), FAP (1:100, #66562, Cell Signaling Technology (CST), USA), EIF4A3 (1:250, ab180573, Abcam), and LC3 (1:200, #12741, CST) was performed at 4 °C overnight. After incubation with the corresponding secondary antibodies (1:2000, ab150077 and ab150083, Abcam) and nuclear staining with 4’,6-diamidino-2-phenylindole (DAPI), the results were observed by the fluorescence microscope (Olympus, Japan). The counting process was conducted by an assessor blind to treatment allocation.

### Conditioned medium collection

The conditioned medium (CM) was collected from CCA cells followed by centrifugation (4 °C, 1000 rpm) for 5 min. After filtration by 0.22 μm filter and concentration using ultra filter unit (Millipore, USA), the collected CM was stored at 4 °C until use.

### Quantitative real-time polymerase chain reaction

Total RNA was isolated using TRIzol reagent (Thermo Fisher Scientific) and then reversely transcribed to generate cDNA using the All-in-One™ First-Strand cDNA Synthesis Kit (GeneCopoeia, USA). Quantitative real-time polymerase chain reaction (qRT-PCR) was carried out using the All-in-One™ qPCR Mix (GeneCopoeia). 2^−ΔΔCt^ method was adopted for calculation the relative level of target genes. Primers are listed in Supplementary Table 2.

### Western blotting

Protein samples were prepared using the NP-40 lysis buffer (Elpis biotech, Korea) supplemented with protease inhibitors. After quantification using the BCA protein assay Kit (Thermo Fisher Scientific), protein samples (30 μg) were subjected to sodium dodecyl sulfate-polyacrylamide gel electrophoresis and blotted onto polyvinylidene fluoride membranes. Next, the membranes were blocked and incubated with primary antibodies against α-SMA (1:1000, ab5694, Abcam), Vimentin (1:1000, ab92547, Abcam), FAP (1:1500, #66562, CST), Smad2 (1:2000, #5339, CST), p-Smad2 (1:1000, #55041, CST), Smad3 (1:2000, #9523, CST), p-Smad3 (1:1000, #9520, CST), KLF4 (1:2000, ab129473, Abcam), EIF4A3 (1:1500, ab32485, Abcam), E-cadherin (1:3000, #3195, CST), N-cadherin (1:1000, #13116, CST), Slug (1:1000, #9585, CST), MMP-2 (1:1500, #40994, CST), Fibronectin (1:1000, ab2413, Abcam), LC3 (1:1000, #43566, CST), p62 (1:1000, ab109012, Abcam), Beclin-1 (1:2000, ab207612, Abcam), and β-actin (1:5000, ab8227, Abcam) overnight at 4 °C. After incubation with HRP-conjugated secondary antibody (1:5000, ab288151, Abcam) for 1 h, immunoreactive bands were visualized using the ECL Kit (Agrisera, Sweden).

### Enzyme linked immunosorbent assay

The production of TGF-β1 and IL-6 was assessed using commercial Human TGF beta 1 ELISA Kit (Abcam) and Human IL-6 ELISA Kit (Abcam), following the protocols, respectively.

### CCK-8 assay

The viability of CAFs or CCA cells was assessed using CCK-8 solution (Sigma-Aldrich). Briefly, 2000 cells seeded in 96-well plates were reacted with 5 mg/mL CCK-8 reagent for 1 h at 37 °C away from light. The results were detected at 450 nm on a microplate reader (Thermo Fisher Scientific).

### Colony formation assay

Cells were seeded onto 6-well plates at a density of 1000 cells per well. After culture for two weeks, the colonies were fixed with 4% paraformaldehyde and stained with 0.5% crystal violet. The number of colonies was counted and analyzed. The counting process was conducted by an assessor blind to treatment allocation.

### Scratch assay

Cells were inoculated into 12-well plates and cultured until confluence. Then, a scratch was created using the 200 μL microtubule tip. After washing, cells were maintained in serum-free medium. Images were taken at 0 h and 24 h after scratch and quantitively analyzed using Image J software.

### Transwell assay

Transwell invasion assay was carried out using Transwell chambers (Corning, USA) pre-coated with Matrigel (Corning). Cells suspended in serum-free medium were seeded in the top chambers and medium containing 10% FBS was added in the lower chamber. After maintenance for 24 h, the invasive cells were fixed in 4% paraformaldehyde and stained with 0.5% crystal violet. The images were photographed under a microscope. The counting process was conducted by an assessor blind to treatment allocation.

### Bioinformatic analysis

The binding of KLF4 to TGF-β1 promoter was predicted by JASPAR database. RNA Interactome database was adopted to predict the interaction between EIF4A3 protein and KLF4 mRNA. The direct interaction between circ_0020256 and EIF4A3 protein was analyzed by Circular RNA Interactome database. The UALCAN and GEPIA databases were utilized for differential expression of EIF4A3 and KLF4 in CCA and its correlation with lymphatic metastasis and survival.

### Dual luciferase reporter assay

The wild type (WT) sequences of TGF-β1 promoter or the mutant (MUT) sequences were inserted into the pGL3 Luciferase Reporter vector (Promega, USA). The luciferase reporter constructs together with OE-NC or OE-KLF4 were transfected into CCA cells. The luciferase activity in cell lysates was detected at 48 h after transfection using the Luciferase Reporter Assay Kit (Abcam).

### Chromatin immunoprecipitation

The ChIP Kit (Abcam) was used to determine the binding of KLF4 to TGF-β1 promoter. In short, cells were received treatment with 1% formaldehyde for 10 min and subsequent glycine for 5 min. Then, cell lysates were subjected to sonication for the generation of chromatin fragments. Immunoprecipitation was performed using anti-KLF4 (1:50, ab215036, Abcam) or anti-IgG (1:50, ab172730, Abcam) antibody overnight at 4 °C. The immunoprecipitated DNAs were purified and evaluated by qPCR.

### RNA immunoprecipitation

The Magna RIP™ RNA-Binding Protein Immunoprecipitation Kit (Millipore) was selected for RIP assay. Briefly, CCA cell lysates were obtained after lysis with RIP lysis buffer. Subsequently, cell lysates were co-precipitated with anti-EIF4A3 (1:50, ab180573, Abcam) or anti-IgG (1:50, ab172730, Abcam) antibody at 4 °C overnight. The magnetic beads were utilized for extraction of RNA-protein complexes. The RNAs were purified by proteinase K and measured by qRT-PCR.

### Fluorescence in situ hybridization

CCA cells were seeded on coverslips and then fixed in methanol/acetic acid solution. Subsequently, coverslips were air-dried and pre-dehydrated in ethanol. The Cy3-labeled probe for KLF4 (Servicebio, China) was denatured for 10 min at 80 °C. Coverslips were incubated at 72 °C for 5 min for denaturation and immersed in ethanol for re-dehydration. Subsequently, coverslips were immersed in hybridization solution and probed with the Cy3-labeled probe for KLF4 at 37 °C for 16 hours. Next day, coverslips were washed, stained with DAPI (Beyotime) and mounted for imaging under a confocal microscope (Olympus). The counting process was conducted by an assessor blind to treatment allocation.

### RNA pull-down assay

The CCA cells were lysed, and cell lysates were collected. The biotin-conjugated sense or anti-sense circ_0020256 probe was mixed well with cell lysates and incubated 4 °C for 4 h. Then, streptavidin-conjugated magnetic beads were added into samples and incubated for additional 2 hours at 4 °C with gentle rotation. Subsequently, fractions pulled down by circ_0020256 probe were eluted, and the abundance of EIF4A3 was examined by western blotting.

### Sanger sequencing and evaluation of RNA stability

To validate the “head-to-tail” back-splicing of circ_0020256, PCR was performed and products cloned into pGEM-T Easy vector (Promega) were subjected to Sanger sequencing. For detection of circ_0020256 stability, the isolated RNAs were treated with 0.4 μL RNase R (Sigma-Aldrich) at 37 °C for 20 min. On the other hand, CCA cells were exposed to 2 μg/mL actinomycin D (Sigma-Aldrich) for various time periods to test the half-lives of circ_0020256 and KLF4 mRNA. Finally, the expression levels of circ_0020256 or KLF4 were assessed by qRT-PCR.

### Xenograft model

Male BALB/C nude mice (nu/nu) (4–6 weeks old) were purchased from SJA Laboratory Animal Co., Ltd. (Hunan, China). The mice were randomly divided into four experimental groups (*n* = 10 per group): OE-NC, OE-circ_0020256, sh-NC, sh-circ_0020256 groups. CCA cells (1 × 10^6^) infected with lentiviruses carrying OE-NC, OE-circ_0020256, sh-NC, or sh-circ_0020256 were subcutaneously injected into the nude mice. The tumor size (length and width) was recorded and tumor volume was calculated using the formula: (length × width^2^)/2. All mice were subjected to euthanasia through cervical dislocation. The xenografts were collected and weighed. The investigators were blinded to grouping assignment. All procedures were approved by the Ethics Committee of the Sixth Affiliated Hospital, Sun Yat-sen University.

### Immunohistochemical staining

Tumors were fixed in 4% paraformaldehyde, embedded in paraffin, and sliced into 4 μm sections. After deparaffinization and rehydration, the sections were treated with hydrogen peroxide for 10 min and then immersed in 5% bovine serum albumin (BSA). Thereafter, incubation with anti-Ki-67 (1:200, ab15580, Abcam), anti-vimentin (1:400, ab92547, Abcam), or anti-α-SMA (1:100, ab5694, Abcam) antibody was performed overnight at 4 °C, followed by application of secondary antibody. The sections were developed with DAB and observed under a microscopy (Olympus). The counting process was conducted by an assessor blind to treatment allocation.

### Statistical analysis

All cell experiments were performed in at least three biological replicates, and each biological replicate contained three technical replicates. Sample sizes of animal experiments (*n* = 10 per group) were based on previous studies using similar analysis of xenograft model [36, 37]. Statistical analyses were carried out using GraphPad Prism 7 software. All the data meet the assumption of normal distribution and presented as mean ± standard deviation (SD). Comparisons were analyzed by one-way analysis of variance (ANOVA) followed by Tukey’s post-hoc test among three or more groups or Student’s *t* test between two groups. The correlation between circ_0020256 and EIF4A3 expression was analyzed by Pearson correlation analysis. *P* < 0.05 was defined to be statistically significant.

## Supplementary information

Supplemental materials

Supporting information for reviewers-STR profiling

## Data Availability

The datasets generated during and/or analysed during the current study are available from the corresponding author on reasonable request. Supplementary Tables 1–2 and original western blots were shown in Supplemental materials.
