# Supplementary material for: Circ_0020256 induces fibroblast activation to drive cholangiocarcinoma development via recruitment of EIF4A3 protein to stabilize KLF4 mRNA
Source: Cell Death Discov. 2023 May 13;9:161. doi: 10.1038/s41420-023-01439-5 (PMC10183031; doi:10.1038/s41420-023-01439-5)

**Supplementary Table 1 Clinicopathological characteristics of cholangiocarcinoma patients.**

| **Clinicopathological characteristics** | **Cases (n)** |
| --- | --- |
| Age (years) |  |
| >60 | 27 |
| ≤60 | 18 |
| Gender |  |
| Female | 25 |
| Male | 20 |
| Lymph node metastasis |  |
| Yes | 31 |
| No | 14 |
| Differentiation |  |
| Low | 29 |
| Medium | 8 |
| High | 18 |
| TNM stage |  |
| I-II | 13 |
| III-IV | 32 |

**Supplementary Table 2 Oligonucleotide primer sets for qPCR**

| **Name** | **Sequence (5’-3’)** | **Length** |
| --- | --- | --- |
| TGF-β1 F | CGACTCGCCAGAGTGGTTAT | 20 |
| TGF-β1 R | CGGTAGTGAACCCGTTGATGT | 21 |
| KLF4 F | ACCCTGGGTCTTGAGGAAGT | 20 |
| KLF4 R | CGGGACTGACCTTGGTAATG | 20 |
| EIF4A3 F | GACTCTGGAAGGCATCAAGC | 20 |
| EIF4A3 R | AGTGAAGTTGGCTTCCCTCA | 20 |
| Circ_0020256 F | AGACCGACTGCCAGTAATAG | 20 |
| Circ_0020256 R | CGGTCTTGGTCAAGTCTTATTC | 22 |
| β-actin F | CCCTGGAGAAGAGCTACGAG | 20 |
| β-actin R | CGTACAGGTCTTTGCGGATG | 20 |

**Original western blots**


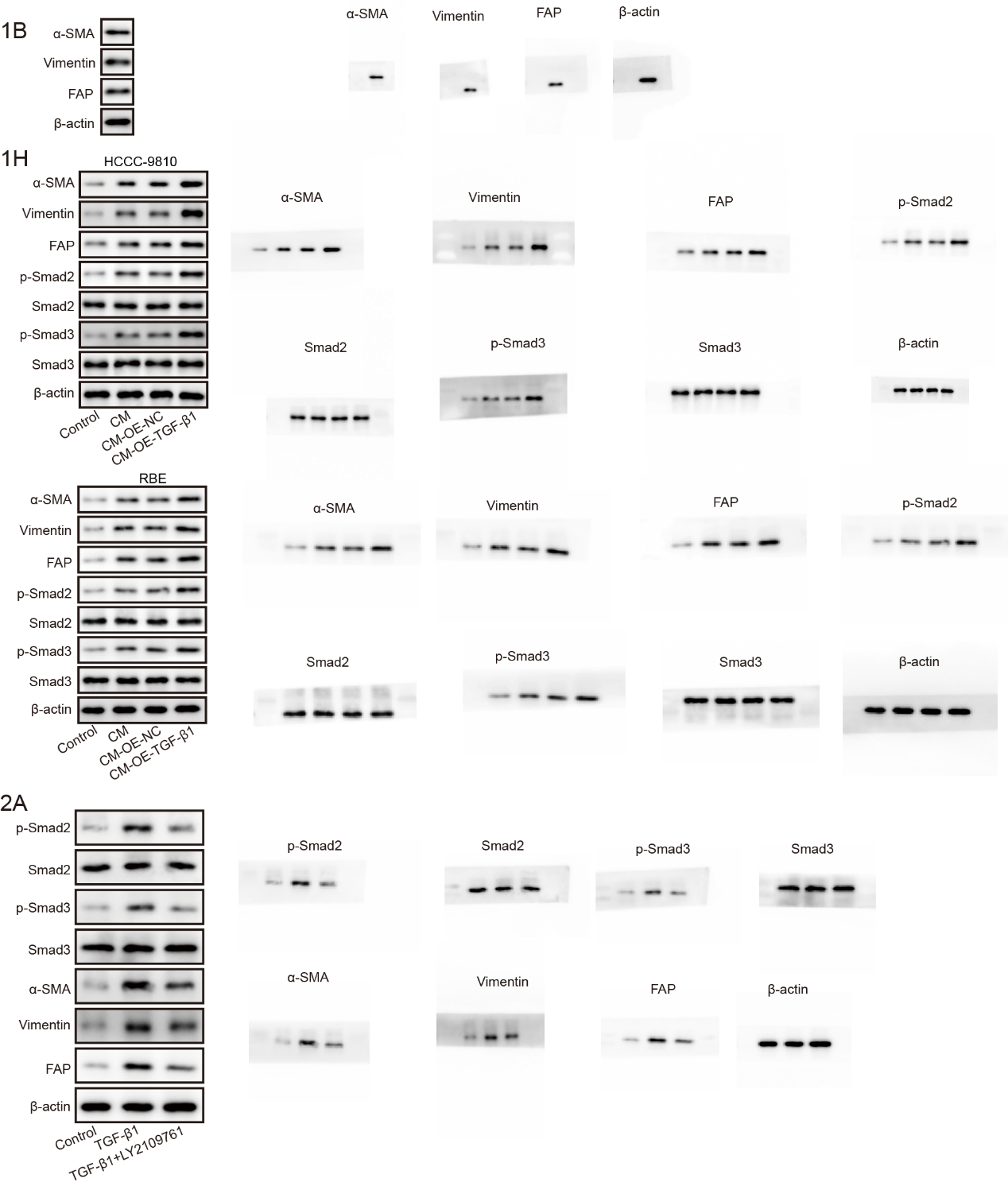

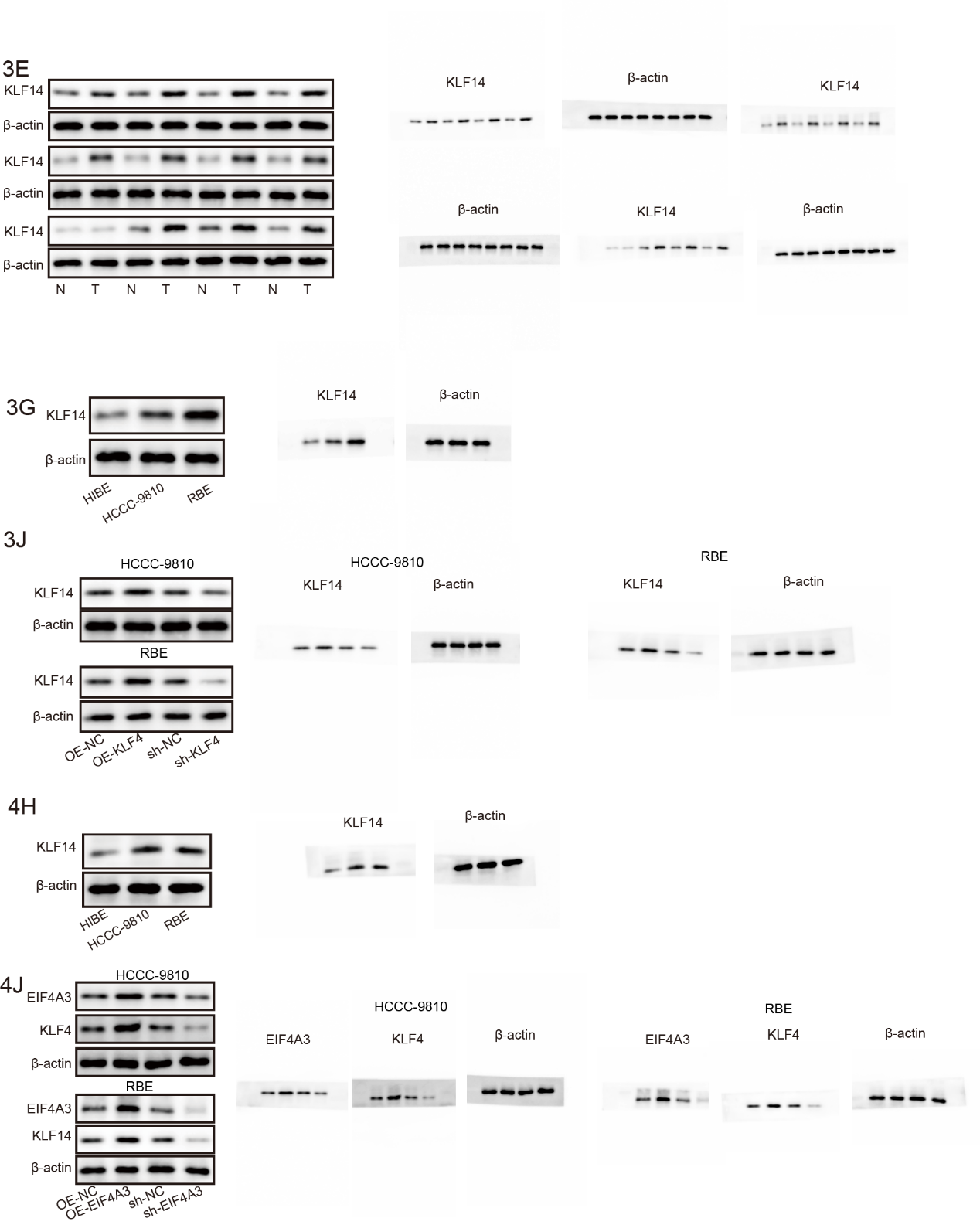

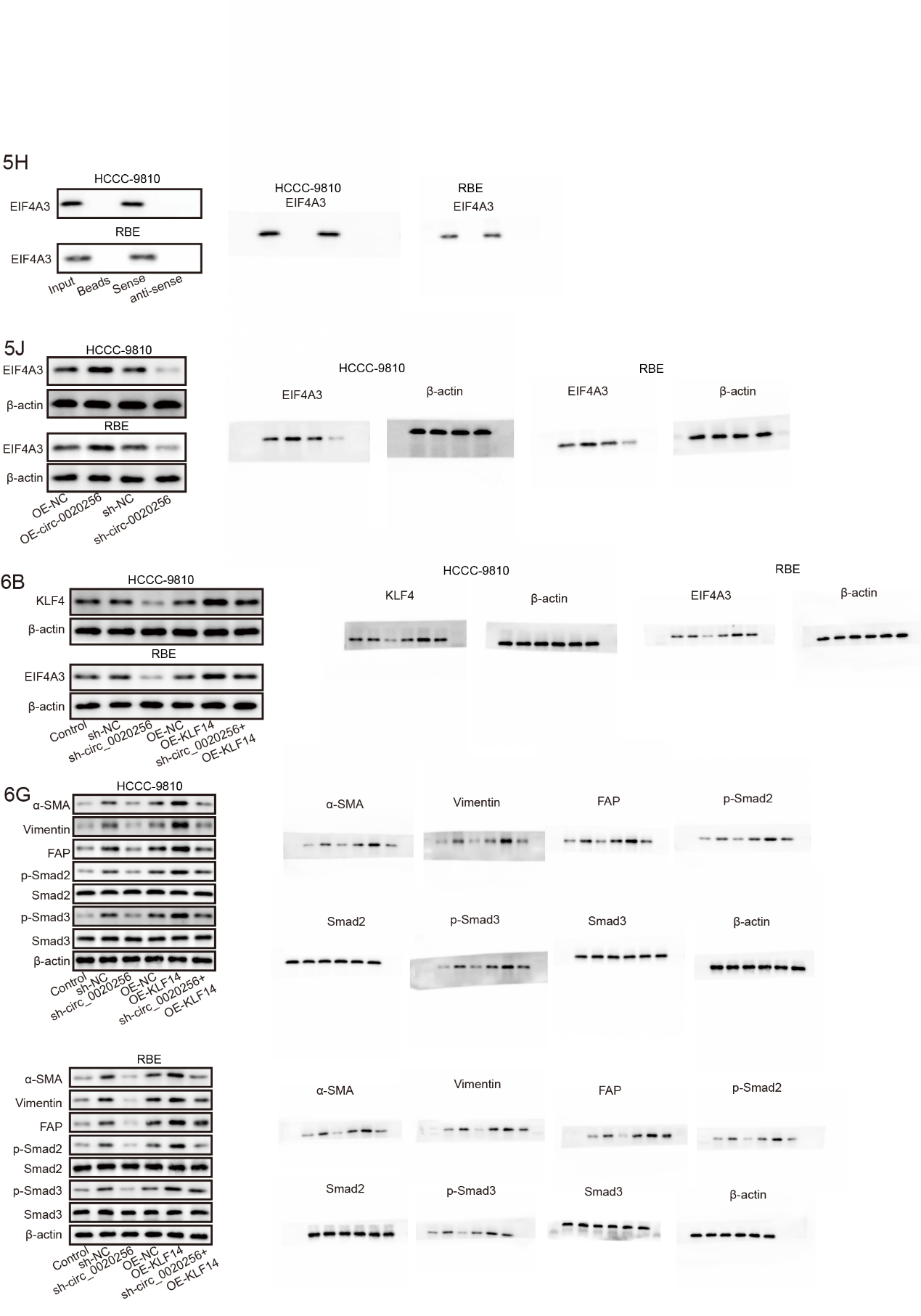

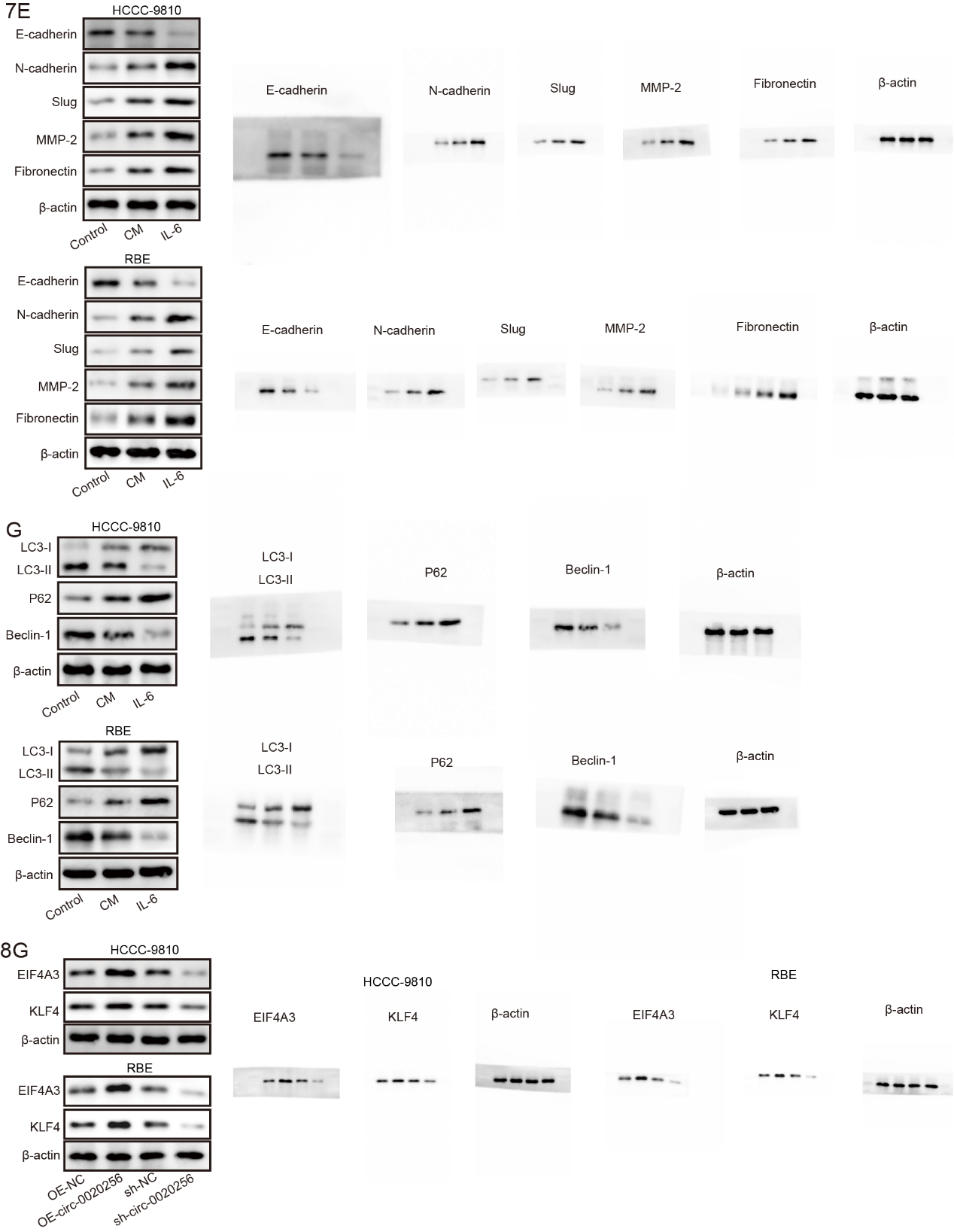

Supplement: Supplementary file 1 — Supplemental materials [file 41420_2023_1439_MOESM1_ESM.docx]
