# Supplementary material for: Circ_0020256 induces fibroblast activation to drive cholangiocarcinoma development via recruitment of EIF4A3 protein to stabilize KLF4 mRNA
Source: Cell Death Discov. 2023 May 13;9:161. doi: 10.1038/s41420-023-01439-5 (PMC10183031; doi:10.1038/s41420-023-01439-5)

**RBE细胞STR鉴定报告**

**样品编号：**

| 客户样本编号 | 公司编号 |
| --- | --- |
| RBE | 20220614-05 |

**样品数量：**1

**样品性状：**细胞系

**检测项目：**STR

**送检单位：**赛百慷

**检测方法：**用Axygen的基因组抽提试剂盒提取DNA，采用20- STR扩增方案扩增，在ABI 3730XL型遗传分析仪上对STR位点和性别基因Amelogenin进行检测。

**检 测 结 果**

1. **检验基本情况**

|  | **多等位基因** | **匹配细胞系** | **细胞库** | **EV值** | **匹配说明** |
| --- | --- | --- | --- | --- | --- |
| 20220614-05 | 无 | RBE | DSMZ | 1.00 | 完全匹配 |

样本基因型检验结果

- 多等位基因指三等位及以上基因现象。
- 本次检测各细胞分型结果良好。

1. **各样本描述**

- 20220614-05：该株细胞DNA分型在细胞系检索中找到**完全匹配**的细胞系，DSMZ数据库显示细胞名为**RBE**，细胞号对应**RCB1292**。本次检测在该细胞系中**没有发现多等位基因**。

**备注：**待测细胞系与收录于ATCC, DSMZ, JCRB 和 RIKEN数据库的细胞系STR数据进行比对，未收录于以上细胞库的细胞系将无法匹配。


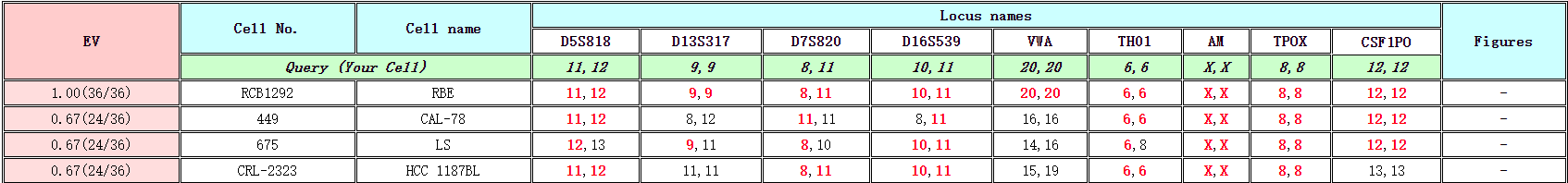


1. **样本分型结果**

| **细胞**20220614-05**的STR位点和Amelogenin位点的基因分型结果** | | | | | | |
| --- | --- | --- | --- | --- | --- | --- |
| Loci | 送检细胞STR信息 | | | 细胞库细胞STR信息 | | |
|  | 送检细胞名：RBE | | | 细胞库细胞名：RBE | | |
|  | Allele1 | Allele2 | Allele3 | Allele1 | Allele2 | Allele3 |
| D5S818 | 11 | 12 |  | 11 | 12 |  |
| D13S317 | 9 | 9 |  | 9 | 9 |  |
| D7S820 | 8 | 11 |  | 8 | 11 |  |
| D16S539 | 10 | 11 |  | 10 | 11 |  |
| VWA | 20 | 20 |  | 20 | 20 |  |
| TH01 | 6 | 6 |  | 6 | 6 |  |
| AMEL | X | X |  | X | X |  |
| TPOX | 8 | 8 |  | 8 | 8 |  |
| CSF1PO | 12 | 12 |  | 12 | 12 |  |
| D12S391 | 18 | 18 |  |  |  |  |
| FGA | 19 | 24 |  |  |  |  |
| D2S1338 | 17 | 24 |  |  |  |  |
| D21S11 | 29 | 29 |  |  |  |  |
| D18S51 | 13 | 15 |  |  |  |  |
| D8S1179 | 13 | 14 |  |  |  |  |
| D3S1358 | 14 | 15 |  |  |  |  |
| D6S1043 | 14 | 14 |  |  |  |  |
| PENTAE | 18 | 18 |  |  |  |  |
| D19S433 | 14 | 15.2 |  |  |  |  |
| PENTAD | 12 | 12 |  |  |  |  |

**其 他 说 明**

1. **分型方案及位点分布**

|  | **方案1** | **方案2** | **方案3** | **方案4** |
| --- | --- | --- | --- | --- |
| 1 | TH01 | TPOX | D3S1358 | AMEL |
| 2 | D12S391 | VWA | D13S317 | D5S818 |
| 3 | D7S820 | D8S1179 | D6S1043 | D2S1338 |
| 4 | CSF1PO | PENTAD | D16S539 | D21S11 |
| 5 | FGA |  | D19S433 | D18S51 |
| 6 | PENTAE |  |  |  |

实验方案及位点

1. **STR数据库比对**

本公司采用DSMZ tools进行细胞系比对，其中包含来自于ATCC, DSMZ, JCRB 和 RIKEN数据库的2455个细胞系STR数据。如果待检测细胞未收录于以上细胞库或这是自行建立的新细胞系将无法进行比对，用户需根据细胞分型结果自行与其他数据库进行比对。

1. **文献引用参考**

1．Authentication testing of HEK 293T and HeLa cell lines have been performed by Shanghai Biowing Applied Biotechnology Co.,Ltd via STR profiling. STR profiles match the standards recommended for HEK 293T and HeLa cell lines authentication

2．AGS, NCI-N87, HGC-27 and HEK293 were STR-authenticated on Dec. 8, 2015 by Shanghai Biowing Applied Biotechnology Co. LTD, Shanghai, China


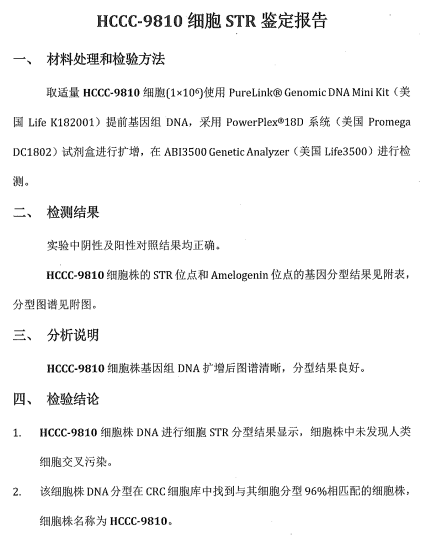


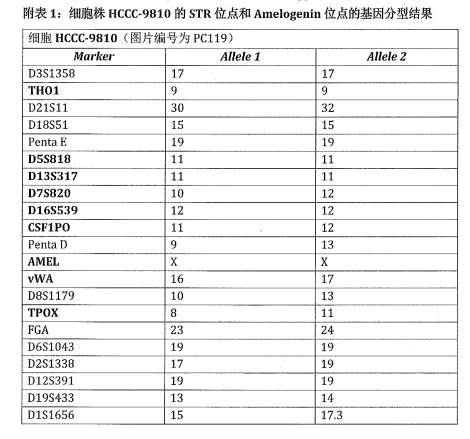


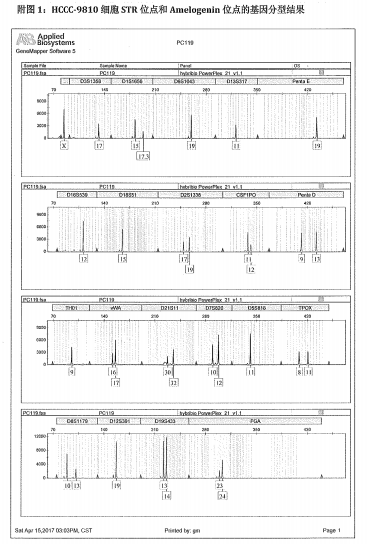

Supplement: Supplementary file 2 — Supporting information for reviewers-STR profiling [file 41420_2023_1439_MOESM2_ESM.docx]
